# Tumor Cell-Derived Exosomes from the Prostate Cancer Cell Line TRAMP-C1 Impair Osteoclast Formation and Differentiation

**DOI:** 10.1371/journal.pone.0166284

**Published:** 2016-11-10

**Authors:** Terese Karlsson, Marie Lundholm, Anders Widmark, Emma Persson

**Affiliations:** 1 Department of Radiation Sciences, Division of Oncology, Umeå University, Umeå, Sweden; 2 Department of Medical Biosciences, Division of Pathology, Umeå University, Umeå, Sweden; Center for Molecular Biotechnology, ITALY

## Abstract

Skeletal metastatic disease is a deleterious consequence of dissemination of tumor cells from numerous primary sites, such as prostate, lung and breast. Skeletal metastases are still incurable, resulting in development of clinical complications and decreased survival for cancer patients with metastatic disease. During the last decade, tumor cell-derived microvesicles have been identified and suggested to be involved in cancer disease progression. Whether cancer exosomes are involved in tumor and bone cell interactions in the metastatic site is still, however, a rather unexplored field. Here we show that exosomes isolated from the murine prostate cancer cell line TRAMP-C1 dramatically decrease fusion and differentiation of monocytic osteoclast precursors to mature, multinucleated osteoclasts. The presence of tumor cell-derived exosomes also clearly decreased the expression of established markers for osteoclast fusion and differentiation, including DC-STAMP, TRAP, cathepsin K, and MMP-9. In contrast, exosomes derived from murine fibroblastic cells did not affect osteoclast formation. Our findings suggest that exosomes released from tumor cells in the tumor-bone interface are involved in pathological regulation of bone cell formation in the metastatic site. This further strengthens the role of tumor cell-derived microvesicles in cancer progression and disease aggressiveness.

## Introduction

Development of clinically significant metastatic disease is one of the most common causes of death in cancer patients. Several cancer forms, including prostate, breast and lung cancer, develop metastases primarily in the skeleton. In prostate cancer, the 5-year survival rate decreases from almost 100% when detected at early stages as localized cancer, to less than 30% with the development of metastatic disease according to statistical measurements by the American Cancer Society [1]. At present, there is no curative treatment available for patients with skeletal metastatic disease. This clearly demonstrates the urgent need for increased knowledge about the cellular communication mechanisms between tumor cells and bone cells resulting in pathological skeletal metabolism in the metastatic site.

Microvesicles are bilayered extracellular vesicles that are released from most cell types and have several functions, such as export of cellular waste and intercellular communication [2]. Exosomes are a subcategory of microvesicles defined as cup-shaped vesicles of 30–150 nm in size, formed by the inward budding of the multivesicular body (MVB) membrane [3]. Exosomes contain bioactive cargo from the cellular cytoplasm, such as proteins, mRNAs and microRNAs [4]. Tumor cell-derived exosomes mirror the characteristics of the cell, and are suggested to play an important role in both tumor growth and disease progression [5]. During the last decade, the role of tumor cell-derived microvesicles in cancer development and progression has received substantial attention. Several reports have been published supporting the role of exosomes as potential prognostic markers and biomarkers for disease detection [6–8]. In addition, exosomal export of drugs, including chemotherapeutic agents such as cisplatin, have been discovered and recognized as part of the cellular characteristics behind acquired treatment resistance [9]. Recent reports have also suggested a role for cancer exosomes both in communication between tumor cells and different cell types in the tumor stroma [10], as well as formation of the pre-metastatic niche [11]. The possible role of exosomes in the pathological communication between tumor cells and bone cells in the skeletal microenvironment is still, however, a rather unexplored field. Here we show that treatment of osteoclast precursor cells with exosomes from prostate cancer cells result in a dramatic decrease in formation of multinucleated, mature osteoclasts.

## Materials and Methods

### Cell lines and cell culture

The murine prostate cancer cell line TRAMP-C1, the murine non-transformed fibroblast cell line MLg and the murine monocytic cell line RAW264.7 were purchased from ATCC/LGC Standards (ATCC numbers CRL-2730, CCL-206, and TIB-71, respectively). All cell lines were used at low passages (maximum +5 passages from purchase) and cultured in basal media as follows: TRAMP-C1 and RAW264.7 cells were cultured in D-MEM with high glucose content (4.5 g/L; Gibco/Life Technologies) and 4 mM stable L-glutamine (GlutaMAX; Gibco/Life Technologies), MLg cells cultured in Eagle’s MEM (E-MEM) containing 2 mM stable L-glutamine (GlutaMAX; Gibco/Life Technologies). All media were supplemented with 10% heat-inactivated fetal bovine serum (HI-FBS, Performance Plus, Gibco/Life Technologies) and 50 μg/mL gentamicin (Gibco/Life Technologies). For culture of TRAMP-C1 cells, 5 μg/mL of bovine insulin (Sigma-Aldrich) and 10 nM dehydroisoandrosterone (DHIA; Sigma-Aldrich) was added to the basal medium. Primary hematopoietic cells isolated from mouse bone marrow were cultured in α-MEM culture medium containing nucleosides, with addition of 2 mM stable L-glutamine (GlutaMAX; Gibco/Life Technologies), 10% fetal bovine serum (FBS, Gibco/Life Technologies), 100 U/mL penicillin, 100 μg/mL streptomycin (Gibco/Life technologies), and 50 μg/mL gentamicin (Gibco/Life Technologies).

### Isolation and characterization of exosomes

For exosome isolation, TRAMP-C1 and MLg cells were cultured with media containing ultracentrifuged HI-FBS to exclude FBS-derived exosomes. Exosomes were isolated from culture supernatants by standard ultracentrifugation as previously described by us and others [12–15]. Briefly, culture supernatant fractions were collected after 48 h and were cleared of cells and debris by serial centrifugations at 3,000 x*g* for 30 min and 10,000 x*g* for 35 min at 4°C. The pellet was discarded and the supernatant was passed through a 0.22 μm filter and ultracentrifuged at 110,000 x*g* for 2 h. The exosome pellet was resuspended in 1 ml of PBS and loaded on a 20–40% sucrose gradient and the ultracentrifugation step was repeated. The exosomes captured in the sucrose layer were collected and washed with PBS. After ultracentrifugation at 110,000 x*g* for 2 h, the exosome pellet was resuspended in PBS and the protein concentration was determined using the BCA protein assay (Pierce) according to recommendations by the manufacturer.

Electron microscopy (EM) of isolated exosomes was performed at the electron microscopy unit Emil, Clinical Research Center, Huddinge, Sweden, to confirm presence of exosomes. Exosome number and size distribution was evaluated by Nanoparticle tracking analysis (NTA; NanoSight N300, Malvern Instruments Ltd.) and samples were diluted 1:500 in PBS before analysis.

### Staining of exosomes and confocal microscopy analysis of exosome uptake by RAW264.7 cells

The cell membrane labeling agent PKH26 (Sigma-Aldrich) was used to label exosomes as described earlier [14]. RAW264.7 cells were seeded at a density of 2x10^4^ cells per well on eight well chamber slides and left to attach overnight before incubation with labeled exosomes (20 ng/10^3^ cells) for indicated periods of time. Cells were fixed with 4% PFA in D-MEM at 37°C for 10 minutes, washed three times with 0.1 M Glycin in PBS, washed another two times in PBS and mounted using ProLongGold antifade reagent with DAPI (Life Technologies). Images were acquired using a Zeiss LSM 710 confocal microscope, and shown are maximum-intensity projections of acquired Z stacks.

### Osteoclastic differentiation of RAW264.7 cells

For studies on osteoclast formation and differentiation, cells from the monocytic cell line RAW264.7 were seeded in 24 well plates at a density of 1,5x10^4^ cells per well. After an initial attachment period of 24 hours, the cells were cultured in the absence (control) or presence of recombinant mouse RANKL (RANKL; 462-TEC, R&D Systems) at a concentration of 2 ng/mL, alone or in combination with exosomes for 96 h. Exosomes from either TRAMP-C1 tumor cells or MLg fibroblastic cells were added at two different concentrations, a higher concentration of 50 ng/10^3^ seeded cells, or the lower concentration of 10 ng/10^3^ seeded cells. After 96 h of culture, the cells were washed twice with PBS, fixed with ice-cold methanol for 10 minutes and stained for tartrate-resistant acid phosphatase (TRAP) using the Leukocyte Acid Phosphatase kit (Sigma-Aldrich) according to the manufacturer’s recommendations. Cells positive for TRAP with ≥ 3 nuclei were considered osteoclasts and counted.

### Proliferation (MTT; 3-[4,5-dimethylthiazol-2-yl]-2,5 diphenyl tetrazolium bromide) assay

For analysis of proliferation, RAW264.7 cells were seeded at a density of 5x10^3^ cells/well in 96-well plates. After initial attachment for 24 hours, cells were cultured for another 72 hours in basal media in the absence (control) or the presence of exosomes at two different concentrations (10 or 50 ng/10^3^ seeded cells). After 72 hours, 10μl of MTT solution from a commercially available MTT kit (Roche) was added to each well and the cells were incubated for an additional 3 hours at 37°C. The following solubilization of the formed formazan crystals was achieved by addition of 100μl per well of 0,1M HCl in isopropanol. Absorbance was read at 550 nm subtracting the background at 650 nm on a Milenia kinetic analyzer spectrophotometer (DPC Diagnostics).

### Total RNA isolation and quantitative PCR analysis

For RNA isolation, RAW264.7 cells were seeded in 12 well plates at a density of 3x10^4^ cells per well. After attachment over night and additional culture in the absence (control) or presence of RANKL (2 ng/mL) alone or in combination with exosomes (10 or 50 ng/10^3^ seeded cells) for 96h, cells were washed twice in PBS and scraped after addition to the wells of Qiazol Lysis Buffer (Qiagen). Total RNA extraction from cell lysates was performed using the miRNeasy mini kit (Qiagen) according to the instructions by the manufacturer. Total RNA was eluted in nuclease-free water and RNA samples were stored at -80°C. For cDNA synthesis, 1.0 μg of total RNA per sample was reverse transcribed using the High Capacity cDNA RT kit (Applied Biosystems) including recombinant moloney murine leukemia virus (rMoMuLV) reverse transcriptase, RNAse inhibitor (1.0 U/ml) and random primers. The cDNA synthesis was carried out using a T-Professional thermocycler (Biometra), and cDNA samples were stored at -20°C.

For quantitative real-time PCR (qPCR) analysis, cDNA samples were diluted 1:5 in nuclease-free water. PCR reactions were carried out in a reaction volume of 10μl (384 well format) and PCR analysis was performed using an ABI PRISM 7900HT Sequence Detection System (Applied Biosystems) and qPCR running protocol as follows: Initial steps at 50° C for 2 min and 95° C for 10 min, followed by 40 cycles of denaturation at 95° C for 15 sec and annealing/extension at 60° C for 1 min. No amplification was detected in control samples where cDNA template was omitted (data not shown). Power SYBRGreen master mix (Applied Biosystems) was used together with the following primers (Invitrogen): β-actin forw: tttgagaccttcaacacccca, rev: gcttctctttgatgtcacgcac; GAPDH forw: actttgtcaagctcatttcc, rev: tgcagcgaactttattgatg; Bax forw: cgggcccaccagctctgaac, rev: acgcggccccagttgaagtt; Bcl-2 forw: tctcaggcccctcgttgcca, rev: accaccgtggcaaagcgtcc; c-Fms forw: gtgcgcagggacagtggctt, rev: tcgagctgctacgtcccggt; Cathepsin K forw: cgtgcagcagaacggaggcat, rev: tacccgcgccactgctctct; DC-STAMP forw: gatcctgccacccgttgccc, rev: cccagtgccagccgcaatca; MMP-9 forw: ccttaccagcgccagccgac, rev: agccggccgtagagactgct; RANK forw: cgagtgctgccgcaggaaca, rev: cctgggcctccttgggtggt; TRAP forw: ctctgaccgtgcccttcgca, rev: gggccactcccaggtctcga. The relative expression of target genes was normalized with reference genes according to the ΔΔCq method, and values are expressed as Ratio target gene/reference gene.

### Isolation and osteoclastic differentiation of primary bone marrow-derived hematopoietic cells

Animal housing and handling was performed according to standards and regulations by the local animal ethics committee at Umeå University (approved ethical application no. A71-15, PI E Persson). Bone marrow was isolated from femurs and tibiae from adult 129/Sv animals after euthanasia by CO_2_ inhalation. Bones were dissected free from surrounding tissues and the bone ends were cut off. Bone marrow was harvested by flushing the marrow cavity with complete α-MEM culture medium, collected cells were passed through a 100 μm cell strainer and erythrocytes were lysed using ACK Lysing Buffer (Gibco). The remaining cells were seeded in culture dishes with non-treated surface (Corning) at a density of 8x10^4^ cells/cm^2^ in complete α-MEM supplemented with recombinant mouse macrophage colony-stimulating factor (M-CSF; 100 ng/mL) to promote proliferation of hematopoietic cells. After an initial culture period of 72 hours, cells were vigorously washed twice with PBS, detached using Versene (Gibco) and re-seeded in dishes with non-treated surface at a density of 5x10^3^ cells/cm^2^ followed by culture for another 72 hours in the presence of M-CSF (100 ng/mL) before initiation of experiments.

Studies on proliferation and osteoclastic differentiation in bone marrow-derived primary osteoclast precursor cells were essentially carried out as described for RAW264.7 cells above, with the exception that basal medium for culture of primary osteoclast precursors was α-MEM supplemented with M-CSF (30 ng/mL).

### Statistical analysis

All data are presented as mean values ± standard error of the mean (SEM) and representative for at least three independent experiments. Statistical analysis was performed using one-way analysis of variance (ANOVA) with Levene’s homogeneity test followed by Dunnett’s 2-sided or Dunnett’s T3 post-hoc test using the statistical software SPSS. A p value of 0.05 was considered significant.

## Results

### Rapid uptake of tumor cell-derived exosomes by osteoclast progenitor cells

**Fig 1:**
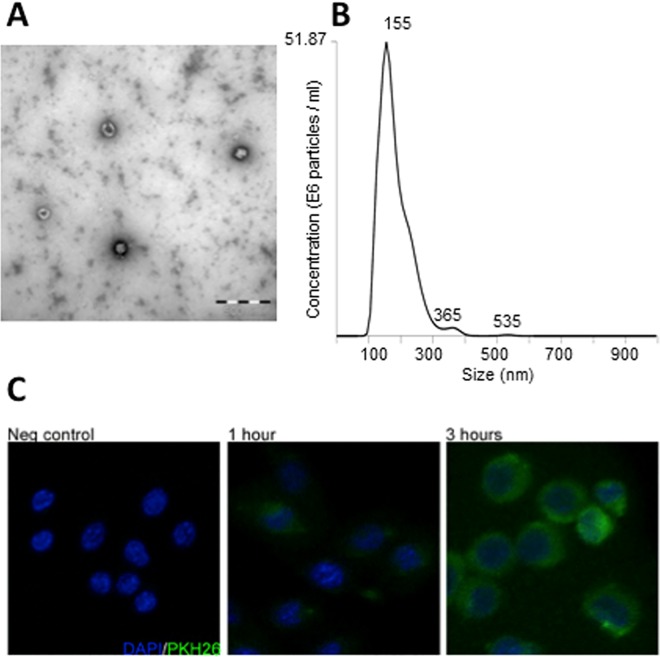
Characterization and cellular uptake of prostate cancer cell-derived exosomes. (A) Representative electron microscopic image of exosomes isolated from the mouse prostate cancer cell line TRAMP-C1, showing the typical cup-shaped morphology. (B) Nanoparticle tracking analysis of isolated exosomes. The calculated size distribution is shown as a mean, peak at 155 nm. (C) Cellular uptake of isolated exosomes by monocytic osteoclast progenitor cells. RAW264.7 cells were seeded on 8 well chamber slides and left to attach overnight before addition of PKH26-labeled TRAMP-C1-derived exosomes at a concentration of 20 ng/10^3^ seeded cells followed by incubation 0–3 hours. Cells were fixed with 4% PFA before mounted using ProLongGold antifade reagent with DAPI. Images were acquired using a Zeiss LSM 710 confocal microscope, and shown are maximum-intensity projections of acquired Z stacks.

For studies on effects by exosomes on bone cell differentiation, exosomes were isolated from the mouse prostate cancer cell line TRAMP-C1. Analysis by electron microscopy revealed the characteristic cup-shape of the isolated exosomes (Fig 1A), and nanoparticle tracking analysis (NTA) confirmed isolated microvesicles being within the expected size distribution (Fig 1B). For detection of uptake of exosomes by osteoclast progenitor cells, exosomes were labeled with the green fluorescent membrane labeling agent PKH26, as described in the ‘Materials and Methods’ section. Murine monocytic RAW264.7 cells cultured on glass chamber slides were treated with exosomes for 0–3 hours, and initial uptake of exosomes by the RAW264.7 cells could be detected already after one hour of incubation by using confocal microscopy (Fig 1C). After three hours, the RAW264.7 cells were densely packed with exosomes, indicating a rapid cellular uptake of exosomes by osteoclast progenitor cells.

### Prostate cancer cell-derived exosomes decrease both proliferation and fusion/differentiation of osteoclast progenitor cells

**Fig 2:**
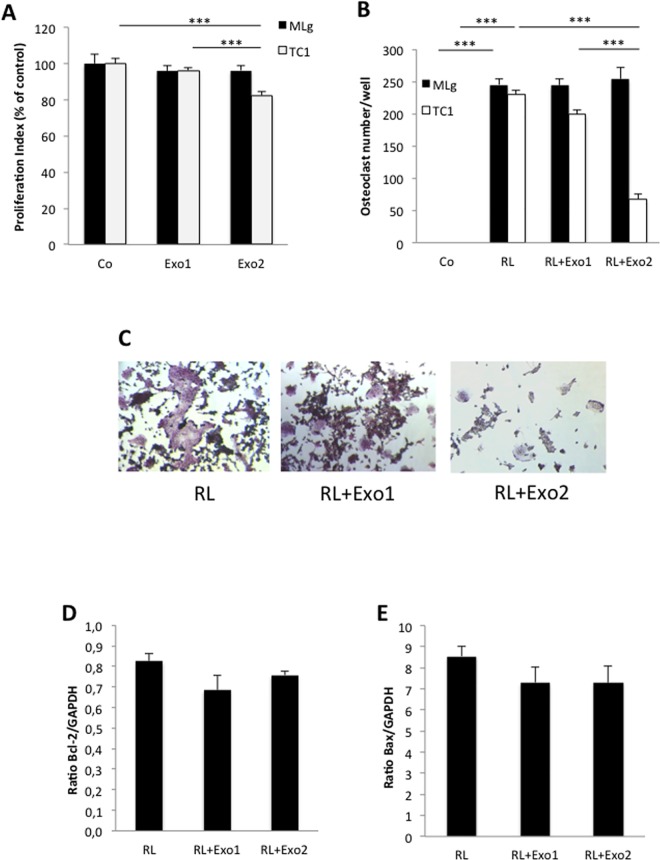
Tumor cell-derived exosomes impair proliferation and osteoclastic differentiation of monocytic RAW264.7 osteoclast progenitor cells. RAW264.7 cells were cultured for 96 hours in the absence or presence of exosomes alone or in combination with the osteoclastogenic factor RANKL (2 ng/mL) before further analysis. Exosomes were added at two different concentrations, either at a lower concentration of 10 ng/10^3^ seeded cells (Exo1) or a higher concentration, 50 ng/10^3^ seeded cells (Exo2). (A) Proliferation (MTT) analysis of RAW264.7 cells after incubation in the absence (Co) or presence of exosomes from fibroblastic MLg cells (black bars) or TRAMP-C1 tumor cells (white bars). Data are obtained from 6 wells per treatment group and presented as means ± SEM, *** *p* ≤ 0.001. (B) Osteoclast formation analysis for RAW264.7 cells treated with RANKL (RL) alone or in combination with exosomes from fibroblastic MLg cells (black bars) or TRAMP-C1 tumor cells (white bars) compared to untreated cells (Co). After 96 h of culture, the cells were stained for tartrate-resistant acid phosphatase (TRAP) using a Leukocyte Acid Phosphatase kit, and cells positive for TRAP with ≥ 3 nuclei were considered osteoclasts and counted. Data are obtained from 4 wells per treatment group and presented as means ± SEM, *** *p* ≤ 0.001. (C) Representative photos of osteoclast cultures after 96 h of treatment with RANKL (RL) alone or in combination with TRAMP-C1-derived exosomes at a concentration of 10 ng/10^3^ seeded cells (Exo1) or a higher concentration, 50 ng/10^3^ seeded cells (Exo2), and subsequent TRAP staining. (D, E) Quantitative PCR analysis of mRNA expression levels for Bcl-2 (D) and Bax (E) in RAW264.7 cells after 96 h of treatment with RANKL (RL) alone or in combination with TRAMP-C1-derived exosomes at a concentration of 10 ng/10^3^ seeded cells (Exo1) or a higher concentration, 50 ng/10^3^ seeded cells (Exo2), before total RNA isolation and PCR analysis. Data are obtained from 3 wells per treatment group and presented as means ± SEM.

Treatment of cells from the monocyte/macrophage cell line RAW264.7 with exosomes isolated from the tumorigenic prostate cancer cell line TRAMP-C1 resulted in decreased proliferation of RAW264.7 cells (Fig 2A). In contrast, exosomes derived from non-transformed, fibroblastic MLg cells did not affect the proliferation rate of RAW264.7 osteoclast progenitor cells (Fig 2A). Differentiation *in vitro* of monocytic RAW264.7 cells to TRAP positive, multinucleated osteoclasts was initiated by addition of the osteoclastogenic factor, receptor activator of nuclear factor κB ligand (RANKL; Fig 2B and 2C). Interestingly, treatment with RANKL in combination with TRAMP-C1 tumor cell-derived exosomes for 96 hours resulted in a statistically significant impairment of RANKL-induced osteoclastic differentiation of RAW264.7 cells treated with the higher concentration of exosomes (50 ng/10^3^ seeded cells; Fig 2B and 2C). Treatment of RAW264.7 cells with RANKL in combination with the lower concentration of TRAMP-C1-derived exosomes (10 ng/10^3^ seeded cells) resulted in formation of osteoclasts smaller in size and with fewer nuclei compared to cells treated with RANKL alone (Fig 2C). The number of osteoclasts was, however, not significantly decreased within this group (Fig 2B). In contrast to the inhibitory effects on osteoclastogenesis induced by exosomes derived from the TRAMP-C1 tumor cells, exosomes isolated from normal fibroblastic MLg cells did not have any effect on osteoclast formation (Fig 2B).

To investigate whether the inhibitory effect by tumor exosomes on osteoclast formation was a result of induction of apoptosis, we analyzed the mRNA expression levels of both the anti-apoptotic marker B-cell lymphoma 2 (Bcl-2) and the apoptotic activator protein Bcl-2-associated X protein (Bax) in RANKL-induced RAW264.7 cells cultured in the absence or presence of TRAMP-C1-derived exosomes. The quantitative PCR analysis showed that neither Bcl-2 nor Bax expression levels were affected by the presence of tumor cell-derived exosomes (Fig 2D and 2E), indicating that the decrease in osteoclast formation seen with tumor cell exosome-treated RAW264.7 cells could not be explained by induction of apoptosis.

### Prostate cancer exosomes decrease expression of osteoclast markers

**Fig 3:**
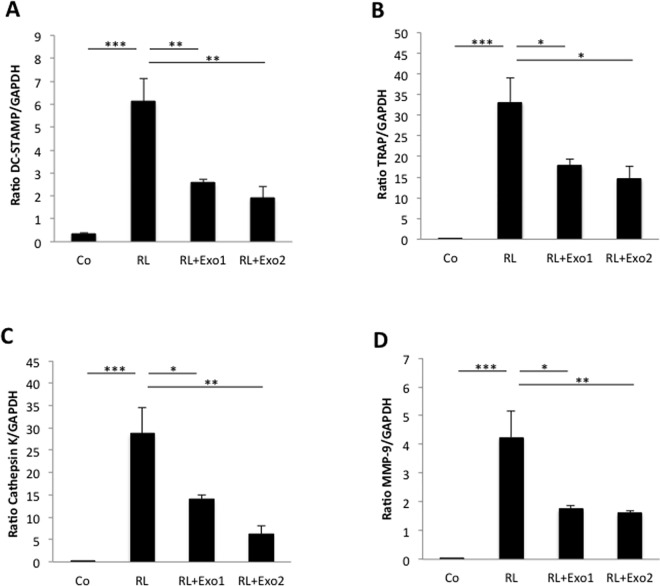
Dedifferentiation of RAW264.7 osteoclasts by tumor cell-derived exosomes. RAW264.7 cells were cultured for 96 hours in the absence (control; Co) or presence of the osteoclastogenic factor RANKL (2 ng/mL; RL) alone or in combination with exosomes from TRAMP-C1 prostate cancer cells at a concentration of 10 ng/10^3^ seeded cells (Exo1) or a higher concentration, 50 ng/10^3^ seeded cells (Exo2), before total RNA isolation and PCR analysis. Quantitative PCR analysis was performed for several markers of osteoclast formation and differentiation, including DC-STAMP (A), TRAP (B), cathepsin K (C), and MMP-9 (D). Data are obtained from 3 wells per treatment group and presented as means ± SEM. * *p* ≤ 0.05, ** *p* ≤ 0.01, *** *p* ≤ 0.001.

Formation of multinucleated, bone-resorbing cells along the osteoclast lineage is a cellular differentiation process strictly regulated by a complex network of both local and systemic molecular factors. To further explore the mechanisms behind the inhibitory effects by prostate cancer cell-derived exosomes on osteoclast formation, possible transcriptional regulation induced by exosomes was analyzed by quantitative PCR. Indeed, incubation for 96 hours of monocytic RAW264.7 cells with the combination of RANKL and TRAMP-C1-derived exosomes resulted in decreased mRNA levels of both the cell fusion marker dendritic cell-specific transmembrane protein (DC-STAMP; Fig 3A) and several markers for osteoclast differentiation and activity, including tartrate-resistant acid phosphatase (TRAP; Fig 3B) and the proteinases cathepsin K (Fig 3C) and matrix metalloproteinase 9 (MMP-9; Fig 3D), compared to cells treated with RANKL alone.

### Prostate cancer exosomes impair osteoclast differentiation of bone marrow-derived osteoclastic precursor cells

**Fig 4:**
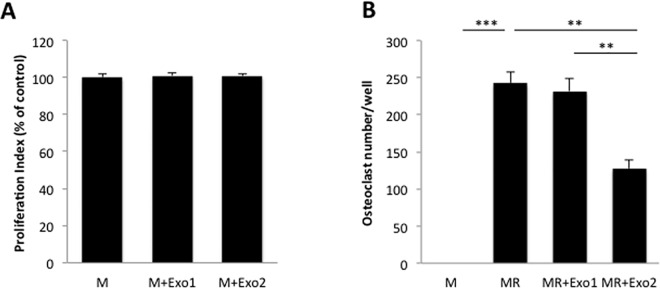
Prostate cancer cell-derived exosomes impair osteoclastic differentiation of bone marrow-derived primary osteoclast progenitor cells. Primary osteoclast progenitor cells were isolated from mouse bone marrow and subsequently expanded *in vitro* for 6 days. Osteoclast precursor cells were then cultured in complete α-MEM culture media supplemented with M-CSF (M; 30 ng/mL) in the absence or presence of TRAMP-C1 prostate cancer cell-derived exosomes alone or in combination with the osteoclastogenic factor RANKL (R; 2 ng/mL) before further analysis. Exosomes were added at two different concentrations, either at a lower concentration of 10 ng/10^3^ seeded cells (Exo1) or a higher concentration, 50 ng/10^3^ seeded cells (Exo2). (A) Proliferation (MTT) analysis of osteoclast precursor cells after incubation in the absence (M-CSF-treatment only; M) or presence of exosomes from TRAMP-C1 prostate cancer tumor cells at two different concentrations (M+Exo1 and M+Exo2, respectively). Data are obtained from 8 wells per treatment group and presented as means ± SEM. (B) Osteoclast formation analysis for bone marrow-derived osteoclast progenitor cells cultured in complete α-MEM culture media supplemented with M-CSF (M) and treated with RANKL (MR) alone or in combination with exosomes from TRAMP-C1 tumor cells at two different concentrations (MR+Exo1 and MR+Exo2, respectively). After 96 h of culture, the cells were stained for tartrate-resistant acid phosphatase (TRAP) using a Leukocyte Acid Phosphatase kit, and cells positive for TRAP with ≥ 3 nuclei were considered osteoclasts and counted. Data are obtained from 4 wells per treatment group and presented as means ± SEM, ** *p* ≤ 0.01, *** *p* ≤ 0.001.

In addition to the studies performed on the monocytic cell line RAW264.7, we also investigated the possible effect by prostate cancer cell-derived exosomes on osteoclast formation and differentiation using primary osteoclast precursor cells isolated from mouse bone marrow. In contrast to the inhibitory effects by tumor exosomes on proliferation of RAW264.7 cells, the primary osteoclast precursor cells did not respond with changes in proliferation rates to the presence of tumor cell-derived exosomes (Fig 4A). However, in line with the inhibitory effects by TRAMP-C1 tumor cell-derived exosomes on osteoclastic differentiation of RAW264.7 cells, treatment of primary osteoclast precursor cells with prostate cancer exosomes also resulted in a distinct decrease in the number of TRAP-positive, multinucleated osteoclasts formed in the presence of the higher concentration of exosomes used (50 ng/10^3^ seeded cells; Fig 4B). Similar to the results obtained for RAW264.7 cells, treatment of primary osteoclast precursor cells with RANKL in combination with the lower concentration of tumor cell-derived exosomes (10 ng/10^3^ seeded cells) did not result in any significant effect on osteoclast formation (Fig 4B).

## Discussion

Development of skeletal metastatic disease is a process regulated by a very complex network of factors released from cells present in the microenvironment of both primary tumor and skeletal tissue. Previous publications have demonstrated that tumor cell-derived exosomes can affect cells at distant sites, thereby promoting disease progression and metastatic development. It was reported by Peinado and colleagues that systemic delivery of fluorescently labeled exosomes from melanoma cells results in exosomal dissemination into both lung and bone tissue, and metastatic development co-localized with exosome deposition sites [11]. In prostate cancer, abundance of microvesicles has also been shown to be higher in metastases than in normal tissues [16]. Our findings presented here show an efficient uptake of prostate cancer cell-derived exosomes by osteoclast progenitor cells, resulting in both decreased proliferation of osteoclast precursor cells as well as dedifferentiation of RANKL-induced osteoclastogenesis seen as a distinct decrease in formation of mature multinucleated osteoclasts. Exosomes isolated from the murine prostate cancer cell line TRAMP-C1 impaired osteoclastic differentiation both in bone marrow-derived primary osteoclast precursor cells as well as in cells from the monocytic cell line RAW264.7. Interestingly, the TRAMP mouse model of prostate cancer from which the TRAMP-C1 tumor cells were originally isolated, has been shown to develop bone metastases with an osteoblastic response [17]. The anti-osteoclastogenic effects by prostate cancer cell-derived exosomes are well in line with the fact that skeletal metastases formed by disseminated tumor cells originating from the prostate are mainly osteoblastic/sclerotic metastatic lesions, characterized by local gain of bone mass [18–20]. Our data suggests that exosomes released by prostate cancer cells might contribute to the osteoblastic phenotype of skeletal metastases in patients with metastatic prostate cancer by suppressing osteoclast formation and thereby also bone degradation in metastatic sites. In contrast to our findings, a recent study by Raimondi and colleagues showed that exosomes derived from multiple myeloma cells increased osteoclastogenesis and expression of osteoclastic markers, including TRAP and MMP-9, in an *in vitro* system similar to ours, also using the RAW264.7 cell line [21]. However, since multiple myeloma-induced bone pathology is characterized by predominantly osteolytic lesions with local loss of bone mass, these findings are also well in line with the clinical situation.

Although skeletal metastases are generally considered either osteoblastic or osteolytic depending on tumor cell origin, phenotypical differences both between and within metastatic lesions are detected in a considerable proportion of patients with metastatic disease. One suggested explanation for the detected heterogeneity is the ‘age’ of the lesions, since initial stages of metastatic establishment is believed to contain an osteolytic phase that allows for expansion of the metastatic site, a phenomenon also suggested to occur in prostate cancer-induced metastases. Whether tumor cell-derived exosomes have different effects on bone cell formation and activity during different stages of metastatic disease is currently unknown and should be investigated further.

Exosomes are biologically stable microvesicles that are frequently found in the circulation. They contain bioactive cargo, including proteins and nucleic acids, which have been shown to function as effectors in recipient cells. Exosomes can affect both differentiation and activity in target cells by cytoplasmic release of exosomal content through either fusion of exosomes to the outer membrane of target cells or by endocytosis [22]. Exosomal exchange between tumor cells and non-tumorigenic cells present in the tumor microenvironment has been shown to both promote and suppress tumorigenesis and disease progression, and thereby also contribute to disease outcome [10, 22, 23]. Furthermore, Melo and co-authors showed in a recent publication that breast cancer-derived exosomes have the capacity to process microRNAs (miRNAs) in a cell-independent manner [24]. The molecular mechanisms behind the inhibitory effect by tumor-derived exosomes on osteoclastogenesis reported here, as well as the different effects seen using exosomes from tumor cells and normal cells are, however, still unknown and needs to be studied further. Investigation and comparison of exosomal content could possibly identify factors responsible for exosome-mediated communication between tumor and bone cells, suggested to contribute to the development of different metastatic bone phenotypes.

In summary, the findings presented here further support a role for tumor-derived exosomes as regulators of bone cell formation in the metastatic site. We show that prostate cancer-derived exosomes impair the formation of osteoclasts, and thereby might contribute to pathological regulation of bone metabolism in skeletal metastatic disease.
